# Mouse Model of Lymph Node Metastasis via Afferent Lymphatic Vessels for Development of Imaging Modalities

**DOI:** 10.1371/journal.pone.0055797

**Published:** 2013-02-06

**Authors:** Li Li, Shiro Mori, Maya Sakamoto, Shoki Takahashi, Tetsuya Kodama

**Affiliations:** 1 Department of Biomedical Engineering, Graduate School of Biomedical Engineering, Tohoku University, Sendai, Japan; 2 Department of Diagnostic Radiology, Graduate School of Medicine, Tohoku University, Sendai, Japan; 3 Department of Oral and Maxillofacial Surgery, Tohoku University Hospital, Sendai, Japan; 4 Department of Oral Diagnosis, Graduate School of Dentistry, Tohoku University, Sendai, Japan; Virginia Commonwealth University School of Medicine, United States of America

## Abstract

Animal studies of lymph node metastasis are constrained by limitations in the techniques available for noninvasive monitoring of the progression of lymph node metastasis, as well as difficulties in the establishment of appropriate animal models. To overcome these challenges, this study has developed a mouse model of inter-lymph-node metastasis via afferent lymphatic vessels for use in the development of imaging modalities. We used 14- to 18-week-old MRL/MpJ−/*lpr*/*lpr* (MRL/lpr) mice exhibiting remarkable systemic lymphadenopathy, with proper axillary lymph nodes (proper-ALNs) and subiliac lymph nodes (SiLNs) that are 6 to 12 mm in diameter (similar in size to human lymph nodes). When KM-Luc/GFP malignant fibrous histiocytoma-like cells stably expressing the firefly luciferase gene were injected into the SiLN, metastasis could be detected in the proper-ALN within 3 to 9 days, using *in vivo* bioluminescence imaging. The metastasis route was found to be via the efferent lymphatic vessels of the SiLN, and metastasis incidence depended on the number of cells injected, the injection duration and the SiLN volume. Three-dimensional contrast-enhanced high-frequency ultrasound imaging showed that the blood vessel volume and density in the metastasized proper-ALN significantly increased at 14 days after tumor cell inoculation into the SiLN. The present metastasis model, with lymph nodes similar in size to those of humans, has potential use in the development of ultrasound imaging with high-precision and high-sensitivity as well as other imaging modalities for the detection of blood vessels in lymph nodes during the progression of metastasis.

## Introduction

Human lymph nodes are round or kidney-shaped organs of the immune system, 2 to 20 mm in diameter, that are distributed throughout the body [1], [2]. Metastasis to regional lymph nodes is an important step in the dissemination of cancer [3], and often occurs at a relatively early stage of tumor development compared with distant metastasis, such as that to the liver and lung [4]. Accurate evaluation of lymph-node metastasis would be very helpful both for tumor staging and for formulation of the most appropriate treatment [5]. Despite the importance of nodal status for many solid malignancies, the methods available to assess lymph nodes are suboptimal [6]. Animal studies of lymph node metastasis are constrained by limitations in the techniques available for noninvasive monitoring of the progression of lymph node metastasis, as well as difficulties in the establishment of appropriate animal models [7].

The two major transplantation models of metastasis currently used are referred to as the spontaneous and the experimental metastasis models [8]. In the former model, tumor cells are injected into an anatomic location (orthotopic implantation), in the tissue from which the tumor had been derived (orthotopic implantation) or in subcutaneous tissue (heterotopic implantation) [8]. In the latter model, tumor cells are directly injected into the circulation (e.g., into the spleen, tail vein or left cardiac ventricle), offering an attractive platform for the study of diagnostic imaging and treatment monitoring [9], [10]. Most lymph node metastasis models are based on the spontaneous metastasis model [11], and these have provided valuable information concerning the biology of lymphatic metastasis [12], [13], lymphatic architecture mapping [14], [15], tumor-cell shedding and trafficking in lymphatic channels [16], and sentinel lymph node mapping [6], [17], [18].

However, the spontaneous metastasis model is limited by its poor performance for the dynamic quantitative assessment of the progression of lymph node metastasis using non-invasive approaches. This is maybe because both the latency period and the initial site of the metastasis vary considerably, and also because conventional imaging modalities are unable to identify changes in the internal structure of lymph nodes, of conventional mice, with a diameter of 1–2 mm [7], [11], [19].

This study describes the development of a mouse model of lymph node metastasis via the afferent lymphatic vessels, which is suitable for use in the development of imaging modalities for dynamic quantitative assessment of metastatic progression. We used MRL/lpr mice developing systemic lymphadenopathy within 3 to 4 months of birth [20], with subiliac lymph nodes (SiLNs) and proper axillary lymph nodes (proper-ALNs) that are 6 to 12 mm in diameter (similar in size to human lymph nodes). The extent of the metastasis to the proper-ALNs after injection of luciferase-expressing tumor cells into the SiLN was evaluated using *in vivo* bioluminescence imaging and three-dimensional contrast-enhanced high frequency ultrasound imaging.

## Materials and Methods

All *in vivo* studies were carried out in strict accordance with the recommendations in the Guide for Proper Conduct of Animal Experiment and Related Activities in Academic Research and Technology, 2006. The protocol was approved by the Institutional Animal Care and Use Committee of Tohoku University (Permit Number: 2010BeLMO-76-20-255, 2009BeA-6, 2010BeA-7). All surgery was performed under sodium pentobarbital anesthesia, and all efforts were made to minimize animal suffering.

### Cell Culture

KM-Luc/GFP cells, which stably express a fusion of the luciferase (Luc) and enhanced-green fluorescent protein (EGFP) genes, were prepared by transfection of MRL/MpTn-*gld/gld* malignant fibrous histiocytoma-like (MRL/N-1) cells [21] (obtained from M. Ono, Tohoku University, on January 24, 2007), using pEGFPLuc (BD Biosciences, Franklin Lakes, NJ, USA) and Lipofectin Transfection Reagent (Invitrogen, Carlsbad, CA, USA). MRL/N-1 cells were established from the spleen of an MRL/gld mouse [22]. We confirmed that KM-Luc/GFP cells have characteristics of malignant fibrous histiocytoma-like cells using histopathologic assessment: there was aggressive growth of pleomorphic fibro-histiocytic cells, with numerous mitotic figures and a storiform pattern (see Suppl. Fig. S1). KM-Luc/GFP cells were cultured in Dulbecco's Modified Eagle Medium (DMEM) supplemented with 10% fetal bovine serum (FBS) containing 1% l-glutamine-penicillin-streptomycin (Sigma-Aldrich, Tokyo, Japan) and 1% Geneticin G418 (Wako Pure Chemical Industries, Ltd., Tokyo, Japan). Before conducting the metastasis experiments, the absence of mycoplasma contamination in the cell cultures was ensured by testing with a mycoplasma detection kit (R&D Systems Inc., Minneapolis, MN, USA).

### Mice

MRL/lpr mice, which develop systemic lymphadenopathy [20], [23], were purchased from the Jackson Laboratory (Bar Harbor, ME, USA) and subsequently bred and maintained at the Institute for Animal Experimentation, Graduate School of Medicine, Tohoku University, Japan. Fifty-eight of these mice (weight, 35 to 45 g; age, 14 to 18 wk) were later used in the experiments. The mean longitudinal diameters of the SiLN and proper-ALN of the mice, as measured using a digital caliper, were 8.4±1.9 mm (mean ± SD; n = 55) and 8.3±1.7 mm (mean ± SD; n = 7), respectively. Of these 58 mice, 42 were used for imaging of metastatic lymph nodes and as negative controls, and 16 were used for detection of the metastatic route.

### Detection of Lymph Node Metastasis

The metastasis model was developed by injecting KM-Luc/GFP cells, suspended in 30 µL PBS, into the SiLN as follows: Group 1: 1×10^4^ cells injected in 1 min (*n = *4); Group 2: 5×10^4^ cells injected in 1 min (*n = *4); Group 3: 1×10^5^ cells injected in 1 min (*n = *4); Group 4: 1×10^5^ cells injected in 2 min (*n = *6); and Group 5: 1×10^5^ cells injected in 3 min (*n = *21). PBS (30 µL) was injected into the SiLN as a negative control (Group 6; *n = *3). Metastasis to proper-ALNs was analyzed using an *in vivo* bioluminescence imaging system (IVIS; Xenogen, Alameda, CA, USA) [24]. Before intraperitoneal injection of luciferin (150 mg/kg; Promega Co., Madison, WI, USA), each mouse was anesthetized with 2% isoflurane (Abbott Japan Co., Ltd., Tokyo, Japan) using an inhalation gas anesthesia system for small laboratory animals. On days 0, 3, 6, 9 and 14 post-inoculation (with the day of inoculation defined as day 0), luciferase bioluminescence was measured (10 min after luciferin injection) for 30 s using the IVIS.

### Identification of Metastasis by *ex vivo* Bioluminescence Imaging

On day 14 post-injection of KM-Luc/GFP cells, luciferin was intraperitoneally injected into each mouse, and 10 min later, the mice were sacrificed by ether inhalation. The ALNs (proper and accessory) and SiLN were surgically removed, and immediately placed in 6-well plates. Bioluminescence was then quantified for 30 s using the IVIS.

### Histological Evaluation

Following *ex vivo* observation of bioluminescence, harvested lymph nodes were fixed overnight in 18.5% formaldehyde in phosphate-buffered solution (PBS), dehydrated, and embedded in paraffin. The embedded specimens were cut into 4-µm-thick serial sections and either stained with hematoxylin and eosin (H&E), or immunostained for luciferase after antigen retrieval. For the latter procedure, sections were incubated for 10 min at 37°C with 0.1% trypsin in Tris buffer, and blocked with 10% rabbit serum (Nichirei Biosciences Inc., Los Angeles, CA, USA) for 10 min at room temperature (RT) to reduce non-specific protein binding. To detect luciferase protein, sections were incubated overnight at 4°C with a goat polyclonal anti-luciferase antibody (ab81823; 1∶1000; Abcam plc, Tokyo, Japan). Immunoreactivity was detected using biotinylated polyclonal rabbit anti-goat immunoglobulin (1∶800; Dako Japan Inc., Tokyo, Japan) for 30 min at RT, peroxidase-conjugated streptavidin (Nichirei Bioscience Inc.) for 30 min at RT, and diaminobenzidine (DAB) as a chromogen in accordance with the manufacturer’s protocol.

### Detection of Luciferase Activity in Organs by Luminometry

To determine luciferase activity in the organs post-inoculation, the inoculated SiLN, the ipsilateral proper- and accessory-ALNs, ipsilateral mandibular lymph node, ipsilateral lumbar aortic lymph node, caudal mesenteric lymph node, contralateral proper-ALN, contralateral SiLN, liver and lungs were surgically removed on day 14 post-inoculation of KM-Luc/GFP cells (Group 3; *n* = 4) or PBS (Group 6; *n* = 3). Tissues were weighed, frozen in liquid nitrogen, homogenized with reporter lysis buffer (Promega Co.), and centrifuged at 12,000×g for 2 min at 4°C. Supernatant samples (10 µL each) were examined for luciferase activity using 50 µL luciferase assay reagent containing D-Lucifer (Promega Co.). Bioluminescence was measured at RT for 10 s using a luminometer (TD-20/20; Turner BioSystems, Sunnyvale, CA, USA). Results are presented in arbitrary units (AUs).

### Visualization of the Metastatic Route

After anesthetization via intraperitoneal injection of 5% sodium pentobarbital (40 mg/kg; n = 15), visualization of the metastatic route was performed using at least 1 of 3 imaging systems: an *in vivo* fluorescence imaging system (IVIS; *n* = 1); an infrared photodynamic camera (PDE; Hamamatsu Photonics K.K., Hamamatsu, Japan; *n* = 12); or a high-speed fluorescence video camera system (HS-FVCS; *n* = 2) comprising a high-power xenon light source (MAX-301; Asahi Spectra Co., Ltd., Tokyo, Japan) and a charge coupled device (CCD) camera (HAS-220; Ditect, Tokyo, Japan) with a lens (ML-Z07545HR; Moritex Co., Tokyo, Japan). The IVIS emitted and detected light at wavelengths of 710 to 760 nm and 810 to 875 nm, respectively, whereas the PDE emitted light at 760 nm and detected light at wavelengths greater than 820 nm. After injection of indocyanine green (ICG; excitation wavelength, 805 nm; emission wavelength, 840 nm; Daiichi Sankyo, Tokyo, Japan) [25] into the SiLN, the IVIS and PDE were used to detect ICG flow in afferent lymphatic vessels from the SiLN to the proper-ALN. A total of 30 µL ICG (125 µg/mL) was injected at speeds of 0.6 mL/h (IVIS, *n* = 1) or 0.5 to 3.0 mL/h (PDE, *n* = 12), using an infusion pump (KDS100; KD Scientific Inc., Holliston, MA). After the PDE had been used to investigate the effect of injection speed into the SiLN on the incidence of metastasis to the proper-ALN (with injection speeds ranging from 0.5 to 1.0, and 3.0 mL/h; *n* = 4 per group), the HS-FVCS was used to determine whether metastasis from the SiLN to the proper-ALN had occurred via the veins or the afferent lymphatic vessels (*n* = 2). Under anesthesia, an arc-shaped incision was made in the abdominal skin from the subiliac to the proper axillary region, and 30 µL of 1.8 µg/µL fluorescein isothiocyanate-labeled bovine serum albumin (FITC-BSA; MW, 70,000; excitation wavelength, 488 nm; emission wavelength, 518 nm; Sigma-Aldrich) was then injected into the SiLN at 1.0 mL/h by means of an infusion pump. A high-power xenon light source with an appropriate filter (MX0490; Asahi Spectra Co., Ltd.) was used to deliver excitation light at a wavelength of 490±2 nm, and a CCD camera with an appropriate filter (MX0510; Asahi Spectra Co., Ltd.) was employed to detect light at a wavelength of 510±2 nm.

To confirm the route of metastasis histologically (*n* = 1), 30 µL Indian ink was injected into the SiLN, and 10 min later, the skin from the subiliac to the proper axillary region, including the veins and afferent lymphatic vessels, was surgically removed and dissected, and the tissue stained with H&E to observe the cross-sectional surface.

### Preparation of Acoustic Liposomes

Acoustic liposomes (ALs) were used as ultrasound contrast agents, and were prepared as described previously [26]. The number of ALs in the lipid solution was calculated to be 3.3×10^12 ^bubbles/mL [27]. The peak diameter of the number distribution was 0.20±0.08 mm, and the zeta potential was −2.40±0.51 mV. Approximately 20% of the ALs contained both liquid and gas, whereas approximately 80% contained liquid alone (i.e., were non-acoustic) [27].

### 3D Reconstruction of the Intranodal Microvasculature in Proper-ALNs using Contrast-enhanced High-frequency Ultrasound with ALs

To reveal changes in the angiogenic vessel density within the metastasized proper-ALNs, contrast-enhanced high frequency ultrasound (CE-HFUS) with ALs was used. Subsequent to injection of the SiLN, over a 1 min period, with either 1×10^5^ KM-Luc/GFP cells in 30 µL PBS (Group 3; *n* = 4) or PBS only (Group 6; *n* = 3), metastasis to the proper-ALN was confirmed by bioluminescence imaging on days 0, 9 and 14. On the same days, a 3D vascular image of the proper-ALN was reconstructed using ALs and a high-frequency ultrasound imaging system (HFUS; VEVO770; VisualSonics, Toronto, Canada) with a 35-MHz center-frequency transducer (RMV-703; axial resolution, 50 µm; focal length, 10 mm) at 100% transmittance power. Mice anesthetized with 2% isoflurane were placed on a stage maintained at 38°C (TM150, VisualSonics) during the scanning period. An ultrasound transmission gel (Parker Laboratories, Inc., Fairfield, NJ, USA) was placed on the proper-ALN, the focal length of the transducer adjusted using a stage control system (Mark-204-MS, Sigma Koki Co., Ltd., Tokyo, Japan), and the working distance of the scan determined by moving the transducer over the proper-ALN using the stage control system.

Generating a 3D dataset requires assessment of multiple 2D ultrasound images that are then reconstructed to achieve the desired 3D image. In our previous experiments [28], we found that the half-life of ALs in solid tumors was 84±12 s when a 100 µL bolus (lipid concentration, 1 mg/mL) was injected manually into the caudal veins of mice within a 10 s period (*n* = 4) [29]. To extend the lifespan of ALs and achieve a longer diagnostic window in the current study, a 200 µL bolus of ALs was injected manually into the caudal vein of each MRL/lpr mouse over a 40 s period, which resulted in an *in vivo* AL half-life of 284±13 s (mean ± SEM; *n* = 3; data not shown). Multiple 2D images of the proper-ALNs were then captured over a period of 156±12 s (mean ± SEM; *n* = 7), shorter than that of the *in vivo* half-life of the ALs, and blending algorithms were used to convert these 2D slices into 3D volumetric images. Blood vessel structures were mapped and reconstructed by tracking the flow of ALs through the vessels, using the software provided with the VEVO 770 system. Regions of interest were drawn manually on every 2D image along the lymph node margins. The blood vessel volumes and densities within the proper-ALNs were calculated using VEVO770 contrast-mode software. Extracted 2D slices were obtained for analysis at 61±5 s (mean ± SEM; *n* = 7).

### Statistical Analyses

All measurements are presented as either the mean ± SD or mean ± SEM. Differences between groups were determined by two-way ANOVA followed by the Tukey-Kramer test. Correlations between *in vivo* and *ex vivo* bioluminescence results were determined using Spearman’s rank correlation coefficient test. Comparisons of metastasis incidence between the various inoculation conditions were performed using Fisher’s exact probability test. A *P* value less than 0.05 was considered to be an indication of statistical significance. Statistical analyses were performed using Excel 2007 (Microsoft, USA) with Statcel2 software.

## Results

### Metastatic Flow and Route

**Figure 1:**
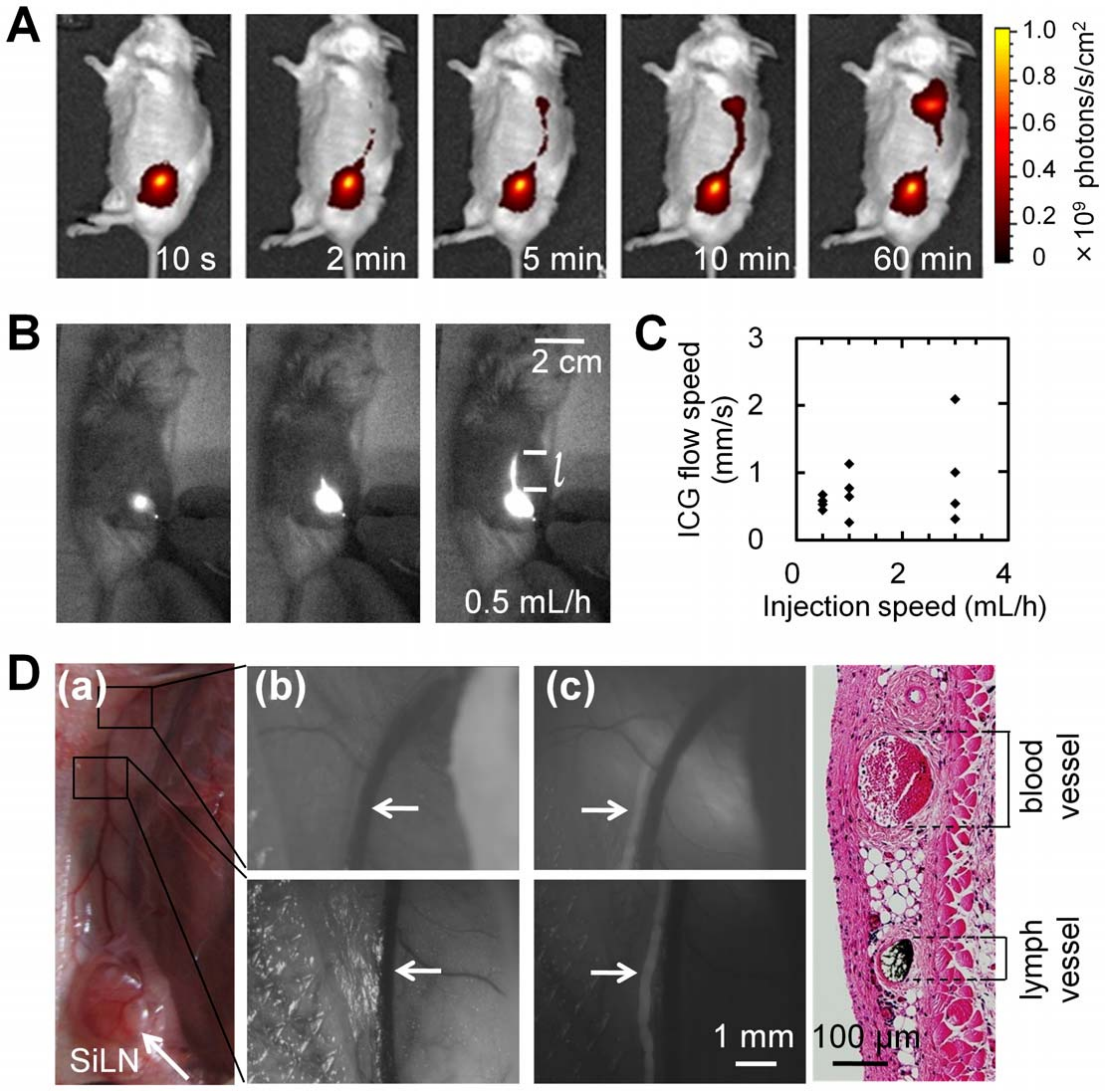
Metastatic flow and route. A. ICG flow from the SiLN to the proper-ALN, observed using an *in vivo* fluorescence imaging system (IVIS; *n* = 1). **B.** Representative PDE images, following an ICG injection speed of 0.5 mL/h. The speed of ICG flow was calculated by dividing the distance, *l*, by the duration of time that had elapsed post-injection. **C.** Graph of the relationship between ICG flow speed and intra-SiLN injection speed (low, 0.5; medium, 1.0; high, 3.0 mL/h; *n* = 4 per group), revealing a low level of variation between individual experiments in the low-speed group. D. HS-FVCS image of the afferent lymphatic vessels after intra-SiLN injection of FITC-BSA solution (*n* = 2). (*a*) Area near the SiLN and proper-ALN captured by a normal digital camera. Two regions of interest were selected. (*b*) Bright field images obtained by HS-FVCS, without use of a fluorescence filter. A thick superficial epigastric vein (→) was observed. (*c*) Fluorescence images obtained by HS-FVCS, with use of an appropriate fluorescence filter (bandwidth: 510±2 nm). A new flow channel filled with FITC-BSA solution (→) appeared at a distance of about 200 µm from the vein. (*d*) Results of hematoxylin and eosin (H&E) staining. The flow channel was identified as the afferent lymphatic vessels by injection of Indian ink. The vein was not stained (*n* = 1).

To identify the metastatic route from the SiLN to the proper-ALN, ICG was injected into the SiLN at 0.5 to 3.0 mL/h (using a syringe pump), and the flow was observed with the IVIS (Fig. 1A) and PDE (Fig. 1B). The ICG flowed via a superficial route at a speed of 0.26 to 2.09 mm/s (Fig. 1C) and accumulated in the proper-ALN of all mice, independent of the injection speed. Flow speed was calculated by dividing the distance, *l*, by the duration of time that had elapsed post-injection (see Supplementary Video S1 and Fig. 1B). Next, the route to the proper-ALN in the abdominal skin flap was investigated (Fig. 1D). To determine whether metastasis had occurred via the veins or the afferent lymphatic vessels, FITC solution was injected into the SiLN and the flow observed using HS-FVCS (Fig. 1D). A thick, superficial epigastric vein was identified under visible light (Fig. 1Db), while the route filled with FITC-BSA solution was identified by fluorescence imaging to be a distance of 200 µm from the vein (Fig. 1Dc). To confirm that this route involved flow via the afferent lymphatic vessels, Indian ink was injected into the SiLN. H&E staining demonstrated that the Indian ink flowed via the afferent lymphatic vessels that connected the SiLN to the proper-ALN (Fig. 1Dd).

### Metastasis in the Lymph Node

**Figure 2:**
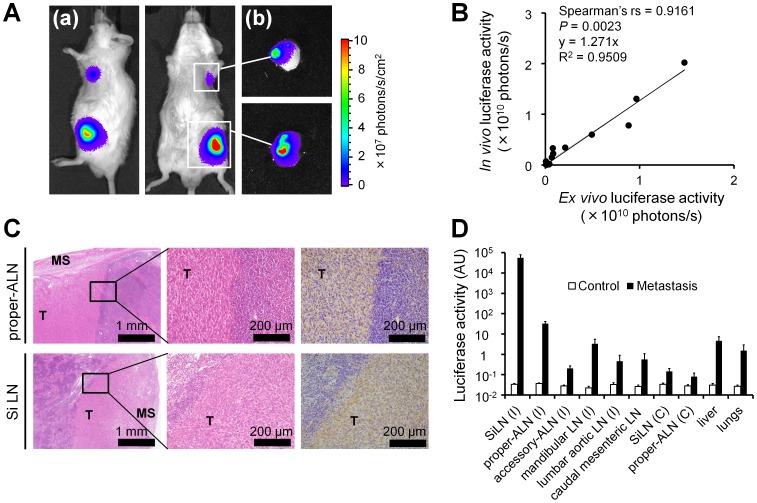
Establishment of the model of lymph node metastasis. A. Representative images captured by *in vivo* bioluminescence imaging of a mouse with tumor cells grafted into the SiLN to promote metastasis to the ALN. (*a*) *In vivo* and (*b*) *ex vivo* bioluminescence signals in the proper-ALN and SiLN on day 14 post-inoculation, indicating that the proper-ALN is the draining lymph node. B. Graph showing the high correlation between *in vivo* and *ex vivo* bioluminescence (*P* = 0.0023; Spearman’s rank correlation coefficient [rs]  = 0.9161; SiLN, *n* = 6; proper-ALN, *n* = 6). C. Results of histological verification. Tumor cells stained with H&E and luciferase-positive immunohistochemical signals in the proper-ALN and SiLN. MS: marginal sinus. T: tumor. D. Dissemination of KM-Luc/GFP cells (metastasis, *n = *4) or PBS alone (control, *n = *3) to each organ, assessed on day 14 post-injection of the SiLN. I, ipsilateral; C, contralateral; LN, lymph node. Error bars indicate the SEM values.

Using IVIS, *in vivo* bioluminescence signals were detected in the ALNs within 3 to 9 days, but were not evident in other organs until day 14 (Fig. 2Aa). *Ex vivo* bioluminescence signals were detected in the proper-ALNs (Fig. 2Ab), but not in the accessory-ALNs (data not shown). A significant relationship was identified between the *in vivo* and *ex vivo* bioluminescence signals (correlation coefficient [rs]  = 0.9161, *P* = 0.0023, *n = *12; Fig. 2B). These results indicate that the *in vivo* bioluminescence signals monitored in the axillary region had been emitted only by the proper-ALNs. H&E and immunohistochemical staining of the excised SiLN and proper-ALN specimens confirmed that the large tumor mass within the SiLN was composed of implanted KM-Luc/GFP cells, and that a metastatic tumor was located in the marginal region of the proper-ALN (Fig. 2C). To further investigate the extent of the systemic metastasis, the luciferase activities in selected organs were measured on day 14 (*n = *4; Fig. 2D). The results showed that metastases had developed primarily in the proper-ALN and to a lesser degree in the adjacent mandibular lymph node, but not significant in the accessory-ALN or other distant lymph nodes. A small number of cells were also detected in the liver and lungs. When PBS alone was injected as a control (*n = *3), the luciferase activity was found to be at background levels.

### Factors Associated with Metastasis

**Figure 3:**
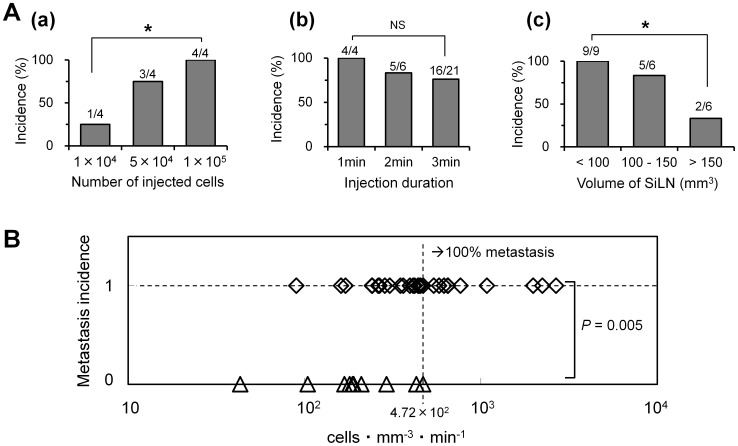
Parameters related to the incidence of metastasis. A. Effects of inoculation conditions on the incidence of lymph node metastasis. Metastatic incidence increased with injection of a larger number cells (a), did not vary significantly with injection duration (b), but decreased with a larger SiLN volume (c). In the values presented above the bars, the denominator represents the number of inoculated mice while the numerator represents the number of mice with metastases in the proper-ALN. NS indicates P>0.05; *P<0.05 calculated by Fisher’s exact probability test. B. Assessment of the correlation between the above 3 parameters and metastasis incidence, using a new parameter, cells mm^−3^ min^−1^. When metastasis incidence was set at 1, 100% metastasis was achieved when the cells mm^−3^ min^−1^ value exceeded 4.72×10^2^ (n = 39). Analyses were performed with the Mann-Whitney U test.

In the current model, cells metastasized from the SiLN to the proper-ALN. To test the hypothesis that the primary parameters related to metastasis are the number of cells injected, the injection duration and the SiLN volume, the effect of each parameter on metastasis was investigated in mice with SiLNs 6 to 12 mm in longitudinal diameter (Fig. 3A). When the injection duration was held constant (1 min), metastatic incidence was found to increase with the number of cells injected (from 1×10^4^ to 1×10^5^ cells), with all 4 mice examined exhibiting metastasis (an incidence of 100%) upon injection of 1×10^5^ cells (Fig. 3Aa). In contrast, metastatic incidence was found to decrease with an increase in the SiLN volume, when the number of cells (1×10^5^) and injection duration (3 min) were held constant. Specifically, the incidence of metastasis was found to be 100% at SiLN volumes less than 100 mm^3^, but significantly reduced at volumes greater than 150 mm^3^ (Fig. 3Ac).

Although the relationship between metastasis incidence and injection duration (1, 2 or 3 min) was found to be not statistically significant, there appeared to be a tendency toward greater metastasis with injections of shorter duration (Fig. 3Ab). To consider the effect of all 3 parameters on metastatic incidence, the new parameter of cells mm^−3^ min^−1^ was introduced; this parameter is directly proportional to the injected cell number and metastasis incidence, while inversely proportional to the injection duration and SiLN volume. It was found that a cells mm^−3^ min^−1^ value greater than 4.72×10^2^ resulted in 100% metastasis induction (*n* = 39; Fig. 3B).

### Progression of Metastasis Assessed using CE-HFUS with ALs

**Figure 4:**
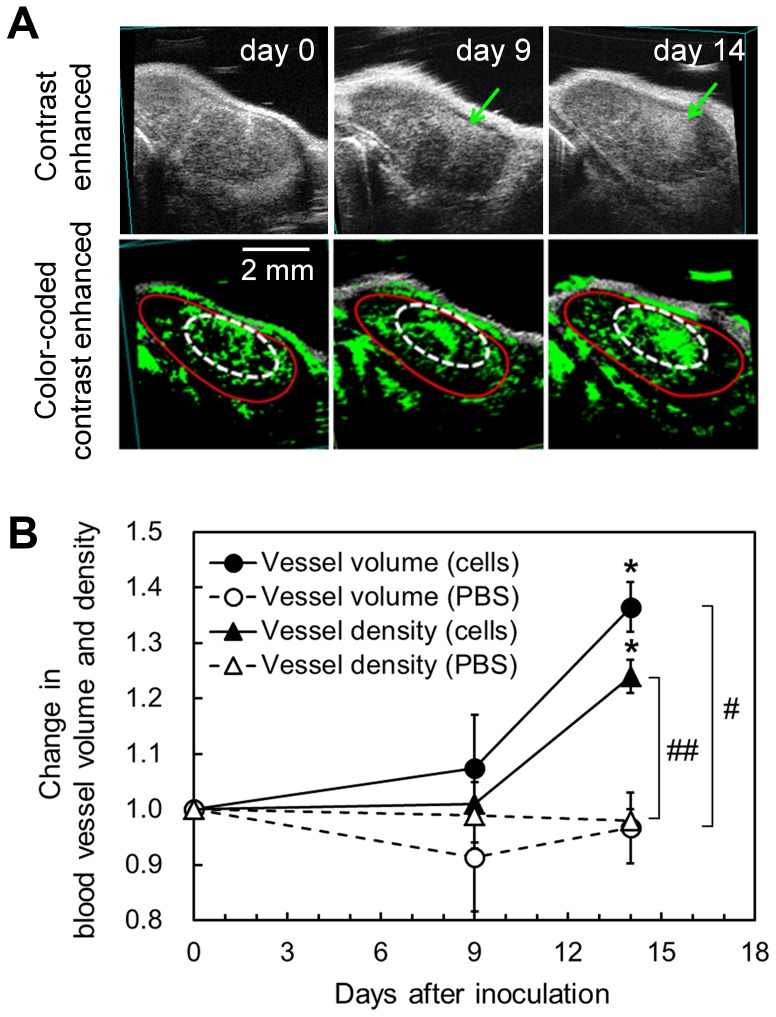
Monitoring of metastatic progression by CE-HFUS with Als. A. Imaging of the temporal changes in angiogenic vessel density in a cross-section of a metastatic proper-ALN, visualized by CE-HFUS with ALs. Blood vessel density increased with tumor progression. Red circles indicate the proper-ALN boundary, arrows and dotted lines indicate the AL-enhanced region, and green highlighting indicates the dense area of neovasculature in the proper-ALN. B. Results of 3D quantitative analysis of the temporal changes in blood vessel volume and density in metastatic proper-ALNs. The term “cells” indicates the metastasized group (1×10^5^ cells/min, *n* = 4) and “PBS” the negative control group (*n* = 3). Values for each group were normalized against the measurement on day 0. Error bars indicate the SEM values. * is for comparison of the temporal change within each group; # is for comparison between groups. * or #, *P*<0.05; ** or ##, *P*<0.01, calculated using two-way ANOVA followed by the Tukey-Kramer test.

CE-HFUS enables real-time reconstruction of co-registered sections into a 3D image that reflects tumor vascularity [28], [30]. Based on this ability, it was hypothesized that CE-HFUS would be suitable for the analysis of cancer progression, and thus could be used to determine whether the proposed mouse model is an appropriate model for the development of diagnostic imaging modalities for lymph node metastasis. Qualitative and quantitative investigations of blood vessel volumes and densities within the metastatic lymph nodes were carried out using CE-HFUS with ALs on days 0, 9 and 14, and the findings were compared with those of the negative control (PBS injection only) group (Fig. 4, A and B). The images were captured within 156±12 s (mean ± SEM; *n* = 7) a time period shorted than the *in vivo* half-life of the ALs (284±13 s; mean ± SEM; *n* = 3) (see Materials and Methods). The period of time over which the images were captured may therefore be sufficiently short to avoid detection of leakage of ALs from vasculature of the proper-ALNs [31]. 2D images extracted from the 3D reconstruction images of the blood vessels demonstrated localized, dense areas of neovasculature (arrows in the upper panels and dotted circles in the lower panels, Fig. 4A), and revealed their development over time. Although it was difficult to confirm that the imaged region was identical for each time point, obtaining 2D vascular-reconstruction images nonetheless enables identification of recognizable remodeling of the vasculature during tumor progression. Since the co-registered sections were not necessarily representative of the entire tumor due to tumor heterogeneity [32], the 3D blood vessel volumes and densities of the metastatic lymph nodes were subsequently investigated quantitatively (Fig. 4B). Values for blood vessel volume and density were normalized against those on day 0. Blood vessel volume significantly increased from 1.00 (day 0) to 1.07±0.10 (day 9) and 1.36±0.05 (day 14) (*P*<0.05); blood vessel density increased from 1.00 (day 0) to 1.01±0.07 (day 9) and 1.24±0.03 (day 14) (*P*<0.05). On day 14, both blood vessel volume (*P*<0.05) and blood vessel density (*P*<0.01) were significantly higher than in the negative control group.

## Discussion

The present study has developed a novel model of lymph-node metastasis, based on the injection of KM-Luc/GFP cells into the SiLNs of MRL*/*lpr mice [20] exhibiting remarkable systemic lymphadenopathy. The importance of this new model is that it is ideally suited for use in the development of imaging modalities to improve the assessment of cancer metastasis to lymph nodes. Mice carrying a lymphoproliferation (*lpr*) mutant gene have defects in the *Fas* gene [33] that result in spontaneous development of an autoimmune lupus syndrome characterized by autoantibody production and massive lymphoproliferation. In this study, the diameter of the lymph nodes was found to be between 6 and 12 mm, which is similar to that of human lymph nodes. The KM-Luc/GFP cell line is a type of transformed MRL/N-1 cell line [21] carrying the *gld* (generalized lymphoproliferative disease) gene, a missense mutation in the *Fas* ligand gene [34]. Since the Fas/Fas ligand axis is relevant to apoptosis [35], apoptosis may not be related to the mechanisms of metastasis in the present model.

The incidence of metastasis in the proper-ALN was found to depend on the number of cells injected, the injection duration and the SiLN volume, with complete (100%) metastasis achieved using cells mm^−3^ min^−1^ values greater than 4.72×10^2^ (Fig. 3B). These findings indicate that increasing the number of cells injected per minute and the internal pressure within the SiLN enhances the efficiency of the formation and survival of cell colonies, resulting in an increase in metastatic incidence [16], [36], [37].

The American Joint Committee on Cancer (AJCC) classifies lymph node metastases on the basis of histological results; isolated tumor cells (cell clusters with diameters ≤0.2 mm), micrometastases (0.2 mm<tumor cell clusters ≤2 mm) and macrometastases (tumor cell clusters >2 mm) [38]. Some studies have revealed a significant relationship between the presence of isolated tumor cells or micrometastases in lymph nodes and poorer prognosis [39], [40], whereas others have failed to find such an association [41]–[43]. However, lymph node macrometastases larger than 2 mm (on the basis of cluster size) have been established to be of prognostic significance [39].

Currently, a lymph node size greater than 10 mm in the short-axis diameter, determined by CT and MRI, is considered to be the most important radiologic criterion for lymph node metastasis [44], [45]. However, this size-based characterization of lymph node metastasis frequently leads to erroneous diagnosis, as when, for example, normal-sized lymph nodes are metastatic or, conversely, abnormally enlarged lymph nodes are solely the consequence of reactive swelling [46]. To improve diagnostic accuracy, it is vital that a new set of diagnostic criteria are established for lymph node metastasis, based on the development and use of high-resolution imaging techniques and specific contrast agents.

As angiogenesis has been found to be an important marker of cancer progression [47], detection of changes in the structures of angiogenesis-induced blood vessels has become a key strategy for the diagnosis of cancer involvement [48]. Based on this finding, the changes in angiogenic vessel volume and density within the proper-ALN were examined using 2D/3D CE-HFUS with ALs [28]. As shown in Fig. 4, significant increases in the angiogenic vessel volume and density in the proper-ALN, compared with the control, were evident at day 14, suggesting that the detection of angiogenesis could be an effective approach to detect lymph node metastasis. In addition, the angiogenesis occurred predominantly after the establishment of metastasis on day 14, and not by day 9. This suggests that angiogenesis may show a period of dormancy during the development of metastasis. Hanahan and Folkman [49] have proposed the concept of an “angiogenic switch” to describe the activation of angiogenesis during the development of a tumor. When a tumor has grown beyond approximately 1 to 2 mm^3^ in volume, it cannot rely on the diffusion of metabolites to meet its needs and therefore must recruit and develop new vessels through the well-known process of angiogenesis [50]. The pattern of the increase in vascularity in the present model is similar to that previously reported [51]. Therefore, the model described here provides an excellent opportunity for the serial quantification and analysis of the changes in blood vessel structure in the lymph nodes, during the progression of metastasis, using non-invasive techniques.

The intravital imaging systems currently used in preclinical cancer research employ magnetic resonance imaging (MRI) at a resolution of 10 to 100 µm, X-ray computed tomography imaging (CT) at a resolution of 50 µm, ultrasound imaging at a resolution of 50 µm, and positron emission tomography at a resolution of 1 to 2 mm [52]. As the size of the mouse lymph node in the present model is similar to that of the human lymph node, the use of this metastasis model to investigate and develop these imaging systems would likely allow for the establishment of new diagnostic methods for diagnosis of metastasis, based on the dynamics of angiogenesis.

In conclusion, we have established a mouse model of inter-lymph node metastasis via afferent lymphatic vessels, using mice exhibiting remarkable systemic lymphadenopathy, with the proper-ALNs and SiLNs 6 to 12 mm in diameter (similar in size to human lymph nodes). This model may be used for the development of highly functionalized, high-resolution ultrasound as well as other imaging modalities for the detection of blood vessels in lymph nodes during metastatic progression.

## Supporting Information

Figure S1 **Malignant fibrous histiocytoma (MFH)-like cells.**
(TIF)

Video S1 **Visualization of the route of lymph node metastasis.**
(AVI)
