# Supplementary figures and images for: Mouse Model of Lymph Node Metastasis via Afferent Lymphatic Vessels for Development of Imaging Modalities
Source: PLoS One. 2013 Feb 6;8(2):e55797. doi: 10.1371/journal.pone.0055797 (PMC3565997; doi:10.1371/journal.pone.0055797)

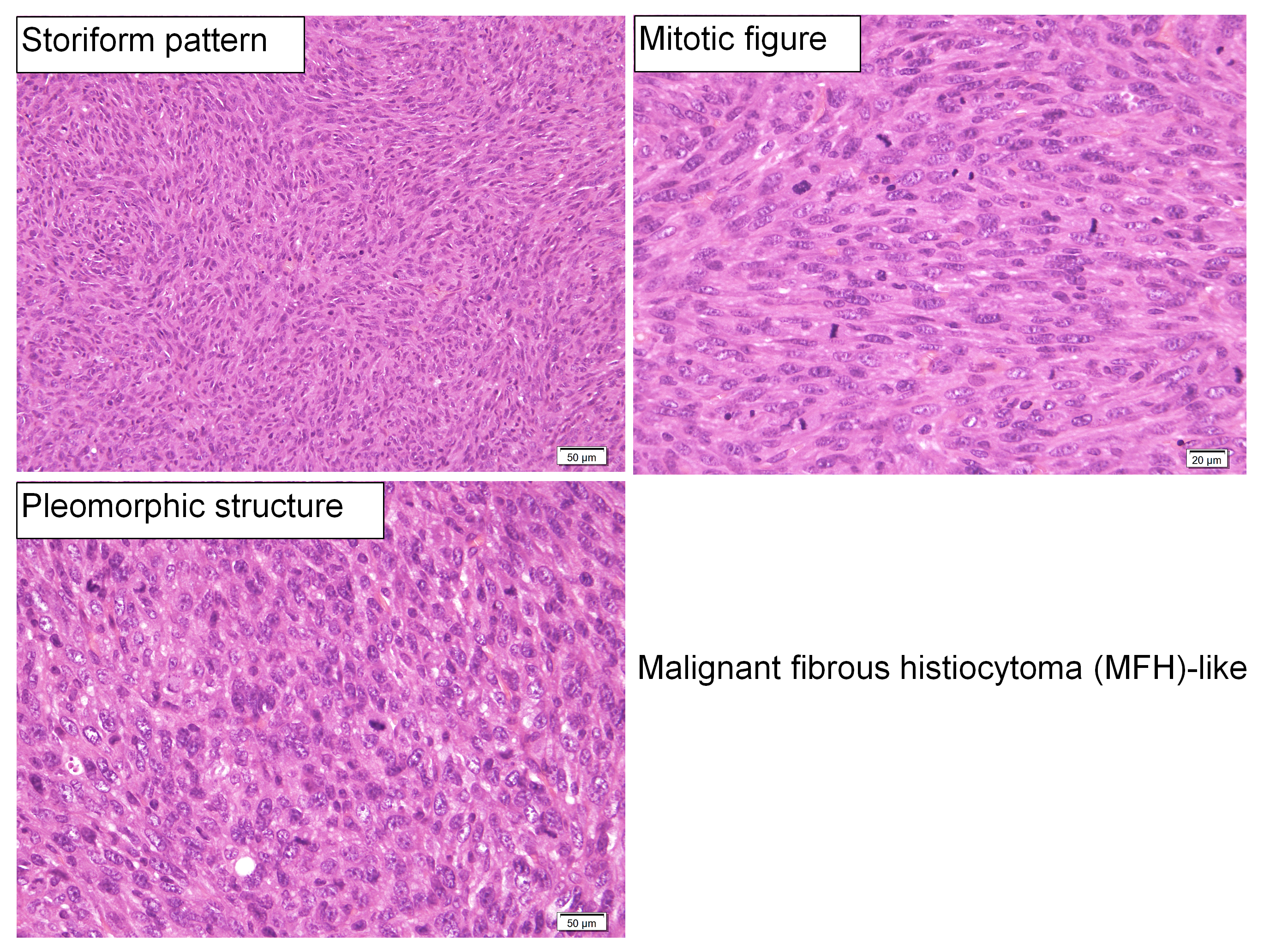

Supplement: Figure S1 — Malignant fibrous histiocytoma (MFH)-like cells. (TIF) [file pone.0055797.s001.tif]
